# Design of a wearable shoulder exoskeleton robot with dual-purpose gravity compensation and a compliant misalignment compensation mechanism

**DOI:** 10.1017/wtc.2024.1

**Published:** 2024-02-12

**Authors:** John Atkins, Dongjune Chang, Hyunglae Lee

**Affiliations:** School for Engineering of Matter, Transport and Energy, Arizona State University, Tempe, AZ, USA

**Keywords:** Design, Exoskeletons, Optimization, Performance Characterization

## Abstract

This paper presents the design and validation of a wearable shoulder exoskeleton robot intended to serve as a platform for assistive controllers that can mitigate the risk of musculoskeletal disorders seen in workers. The design features a four-bar mechanism that moves the exoskeleton’s center of mass from the upper shoulders to the user’s torso, dual-purpose gravity compensation mechanism located inside the four-bar’s linkages that supports the full gravitational loading from the exoskeleton with partial user’s arm weight compensation, and a novel 6 degree-of-freedom (DoF) compliant misalignment compensation mechanism located between the end effector and the user’s arm to allow shoulder translation while maintaining control of the arm’s direction. Simulations show the four-bar design lowers the center of mass by 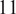 cm and the kinematic chain can follow the motion of common upper arm trajectories. Experimental tests show the gravity compensation mechanism compensates gravitational loading within 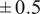 Nm over the range of shoulder motion and the misalignment compensation mechanism has the desired 6 DoF stiffness characteristics and range of motion to adjust for shoulder center translation. Finally, a workspace admittance controller was implemented and evaluated showing the system is capable of accurately reproducing simulated impedance behavior with transparent low-impedance human operation.

## 1. Introduction

Numerous studies have shown that physically strenuous work environments lead to a greater risk of developing musculoskeletal disorders in workers (Ezugwu et al., 2020; Keir et al., 2021). Risk factors for these injuries often come from work involving highly repetitive manufacturing tasks, heavy lifting, and maintaining difficult or awkward postures (Roquelaure et al., 2009). The underlying causes of these injuries have been shown to be linked to work-related highly repetitive motions, and the symptoms and their severity are exacerbated by heavy, sustained loading to the worker’s joints, particular to the shoulder and neck (Andersen et al., 2002).

Studies have shown that the implementation of methods to reduce the forces to the worker’s joints can reduce fatigue and therefore slow or potentially stop the progression of these injuries (Qin et al., 2014). This suggests that robotic systems such as exoskeletons that can assist a wearer’s motion and therefore reduce muscle fatigue and external joint forces could be a viable option for reducing pain and injury for a large portion of the workers in fields such as manufacturing or construction.

Many exoskeletons have been designed and tested for the purpose of assisting workers (Gopura et al., 2016; Crea et al., 2021) and they can largely be divided into the two categories of active and passive exoskeletons. Active exoskeletons often feature a large number of actuators to allow the system complete external control of the exoskeleton to achieve assistance tasks. These systems offer significant flexibility in available movement as well as control schemes at the cost of weight, size, computational complexity, and power consumption (Mallwitz et al., 2015; Kim and Deshpande, 2017). Passive exoskeletons feature little to no active control, and typically relying on spring-driven mechanisms to provide partial gravity compensation over a smaller range of motion and for smaller amounts of net assistance at the cost of controllability and adaptability (de Looze et al., 2016; Hyun et al., 2019; Pacifico et al., 2020). Both approaches to address this problem have their advantages and disadvantages, and the best choice is likely dependent on the specific task or work environment.

At the intersection of active and passive is a design which takes on the characteristics of both active and passive exoskeletons in order to function well in a larger variety of environments, often referred to as semi-active or hybrid exoskeletons (Stewart et al., 2017). Designs using this approach feature a mix of actively controlled actuators and passive components that offer reduced complexity and lighter weight while also providing more assistance than a purely passive design (Gull et al., 2021; Missiroli et al., 2022a).

A compatible assistive controller to provide the desired assistance is crucial for both active and hybrid exoskeletons. While specific details of the controllers are as varied as the exoskeletons themselves, many share commonalities in that they all aim to minimize user effort through a mix of active gravity compensation and feedforward compensation of expected dynamic torques (Gull and Bai, 2020). Many recent hybrid exoskeletons such as the Stuttgart Exo-Jacket (Ebrahimi, 2017) and the hybrid extension of the MATE exoskeleton (Missiroli et al., 2022b) accomplish this through online estimation of the user’s joint torques and the arm’s gravitational load by a dynamic model and active sensing of user behavior which generates a reference compensation torque. This reference torque is then tracked through a lower level admittance or torque controller that serves to follow the user while providing continuous compensation of user effort to provide assistance.

This paper will focus on the design of a hybrid upper-body exoskeleton designed to serve as a platform for assistive controllers that can be used to mitigate the severity of work related musculoskeletal disorders in workers that features a mix of active and passive components. The paper begins with an overview of desired system requirements that inform the design process as a whole followed by a description and analysis of the kinematic chain. Then an overview of major components such as the four-bar method for gravity compensation, the spatial compliant alignment mechanism (SCAM), and the controller design is given. Finally, a set of validation experiments is described that validate each major component individually before testing the system as a whole to show accurate replication of desired admittance controller behaviors and transparent actuation with a human wearer.

## 2. System design

### 2.1. Design requirements

A set of high-level design requirements that the system must achieve in order to properly operate for the intended purpose is:

- The kinematic chain must be chosen such that the workspace of the exoskeleton covers a wearer’s natural range of motion of the upper arm during common motion tasks. Boundaries of this range would cover where the user’s arm is resting at their side, or 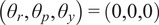 rad, where the user’s arm is extended in front of the body, or 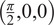 rad, and where the user’s arm is raised to the side of the body at 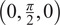 rad.
- The actuators must be independently capable of quickly following the arm and supporting the arm’s weight but not capable of overpowering the user for safety in case of control failure. This corresponds to an actuator with stall torque less than 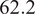 Nm and capable of nominal speed of over 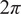 rad/s (Baillargeon et al., 2022).
- The center of gravity of the exoskeleton must be close to the wearer’s natural center of gravity to ensure the system does not alter the wearer’s natural balance as well as to reduce any additional torque on the lower back during bending of the trunk (Waddell and Burton, 2001).
- The gravitational loading due to the weight of the exoskeleton must not be transferred to the wearer’s arm and must be compensated to reduce fatigue from wearing the robot. Additionally, the exoskeleton should partially compensate for the wearer’s arm weight to provide partial passive assistance (Hull et al., 2020).
- The interface between the exoskeleton and the wearer’s arm must include misalignment compensation as to not impede the shoulder joint’s natural translational movement and to reduce any possible joint discomfort (Näf et al., 2018).
- The controlled system must be dynamically transparent at low simulated impedances to not tire the wearer in passive following or low assistance tasks (Zimmermann et al., 2020).

### 2.2. Kinematic chain

**Figure 1.**
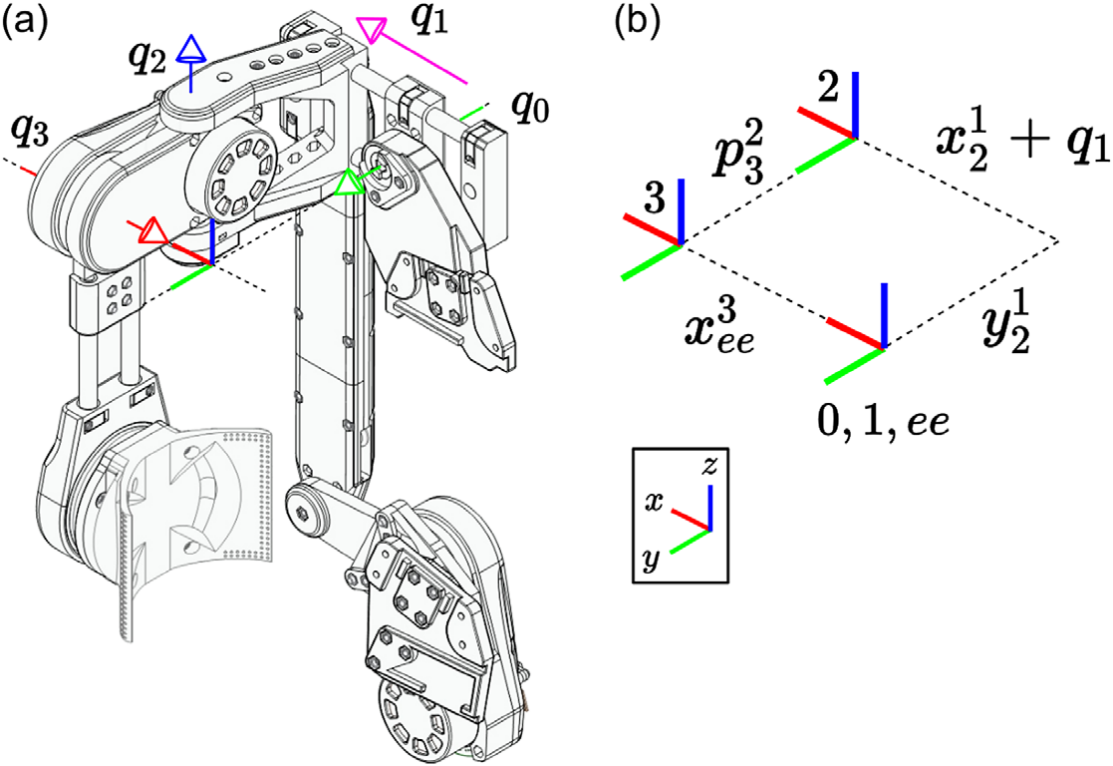
(a) The proposed kinematic chain features a total of four joints, two active and two passive. The first joint is active and allows control of arm abduction and adduction and it is followed by two passive joints. The first is a passive translational joint that is locked after being fit to the wearer, and the second is a passive rotational joint that directs the final active joint to follow the arm. The final active rotational joint allows control of the arm flexion and extension. Together this mix of active and passive joints allows for control of the arm’s direction and passive adjustment to minimize rotational misalignment. (b) Reference frames for the kinematic chain’s inverse kinematics implementation.

The kinematic chain was designed to allow the exoskeleton to follow the highly complicated human shoulder joint with minimal complexity by ensuring active control of the direction of the shoulder while allowing passive joints to compensate for misalignment between the exoskeleton’s end effector and the wearer’s shoulder joint (Figure 1). The first active rotational joint, 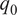 , is placed to support arm abduction and adduction, followed by a translational joint, 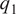 , that is manually adjusted up to 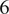 cm to properly fit the wearer and then locked in place, and then the passive rotational joint 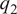 is placed to allow the final axis 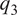 to move with the arm and partially support abduction and adduction, allowing for a mobile instantaneous center of rotation that closely follows the shoulder’s true moving center of rotation.

The forward kinematics as a function of joint angle vector 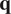 for the given kinematic chain can be described using homogeneous transformation matrices as
(2.1)

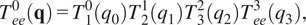

where
(2.2)

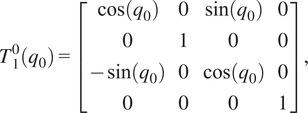



(2.3)

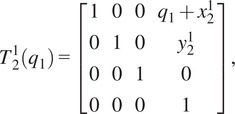



(2.4)

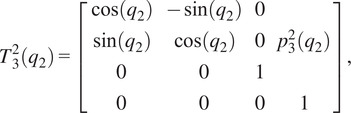



(2.5)

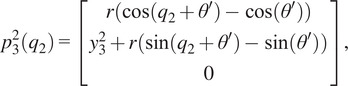



(2.6)

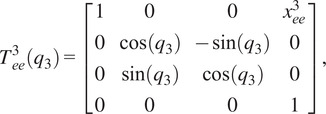

with 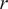 being the distance from the origin to the axis for joint 2, or 
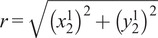
 , and 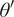 being the angle between the 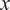 -axis and the direction of the common normal of the 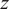 -axis and the passive joint axis corresponding with 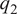 . For the given joint ranges of 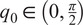 rad, 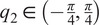 rad, and 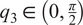 rad, the forward kinematics show that the reachable upper arm directions cover all poses with the arm in front of the user as is requirement for the desired kinematic chain (Figure 2).

**Figure 2.**
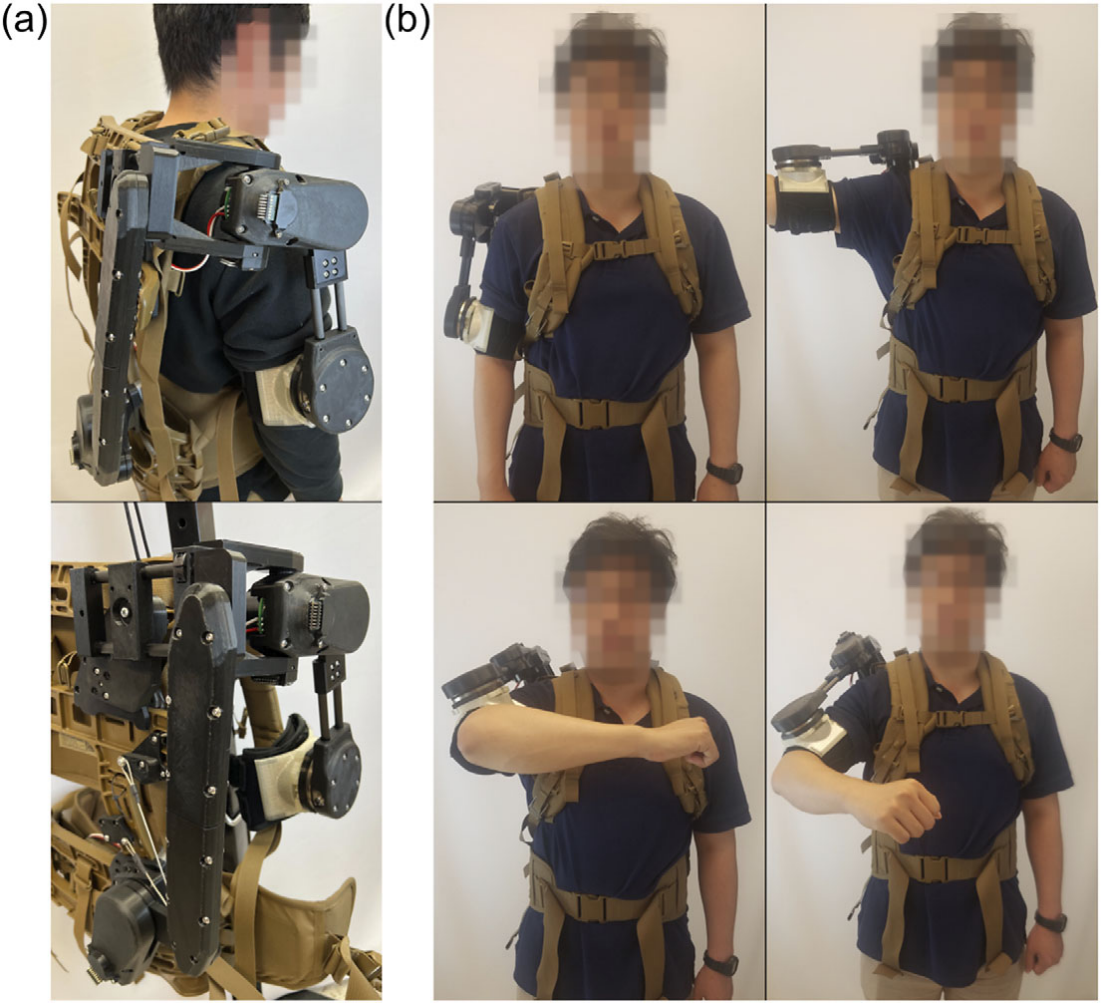
Worn prototype of the final design. (a) Back views of the exoskeleton. Top: Worn exoskeleton in the default configuration. Bottom: Unworn exoskeleton in the default configuration supported by full gravity compensation. (b) Front views of the exoskeleton being worn. Top Left: Exoskeleton is at default configuration with the wearer’s arm resting to their side. Top Right: Arm is extended directly to the side corresponding to 
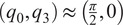
 rad. Bottom Left: Arm is extended in front of the user. This pose corresponds to an active joint configuration of 
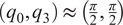
 rad. Bottom Right: Arm is at a natural posture in front of the body and would correspond to an active joint configuration of 
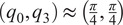
 rad. The person in the figure gave permission for the use of his image.

An advantage of the choice of kinematic chain is the existence of a computationally efficient closed-form inverse kinematics solution. The joint values for all rotational joints can be derived from the rotation matrix of the end effector by noting the angle between the 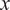 -axis component of the rotation matrix and the 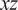 _-_plane corresponds directly to 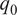 , and similarly the ratio of the 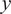 -axis components of the 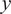 - and 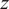 -axes of the rotation matrix corresponds to 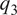 . It should be noted the following inverse kinematics equations are valid when the joint values are within the expected range of 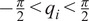 rad, for 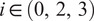 , but similar relations can be derived for other quadrants. These can be computed from 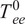 as
(2.7)

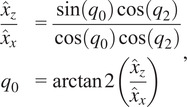



(2.8)

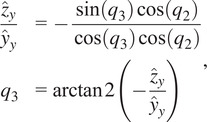

where 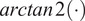 is the four-quadrant arctangent function. It should be noted that this formulation would approach a singularity as 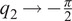 or 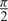 rad; however, these configurations are unreachable when the device is worn by a human wearer.

The passive axis joint value of 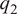 can be simply computed by taking the angle between the rotation matrix’s 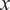 -axis’ 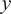 component and the origin frame’s 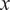 -axis as
(2.9)

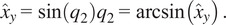



Finally, the translational joint value used for adjusting the exoskeleton to the wearer, or 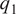 , is known and measured for each user and therefore does not need to be repeatedly computed, but the value can be extracted from the translational 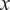 -axis component of the final transformation matrix after the other joints are known by
(2.10)

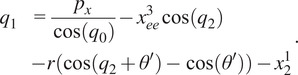



The geometric Jacobian can be derived from the forward kinematics by computing how the joints’ screw axes transform as a function of the jointspace and organizing them into a matrix.
(2.11)

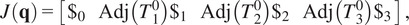

where 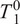 , 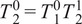 , and 
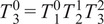
 are all functions of 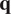 as described in Equations (2.2)–(2.4) and 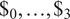 are the 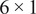 joint unit screw axes (Davidson and Hunt, 2004) in the default exoskeleton configuration (Figure 1). The 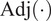 function is the 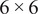 adjoint representation of a transformation matrix (Lynch and Park, 2017).

Since the primary concern of the exoskeleton is the end-effector orientation and corresponding arm direction, a simpler representation of the angular Jacobian, 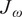 , can be taken from the top three rows of the full Jacobian and removing the second column corresponding to 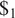 as that axis does not affect the rotation as
(2.12)

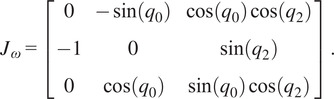



This simple form of the angular Jacobian is easy to invert and allows fast conversion between workspace and jointspace velocities that are used in motor commands, but it also reveals a singular configuration of 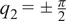 rad as 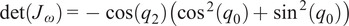 corresponding to alignment of 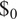 and 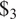 . However, with a human wearer, the joint position of 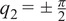 rad would be unreachable and this singular configuration can be safely ignored.

**Figure 3.**
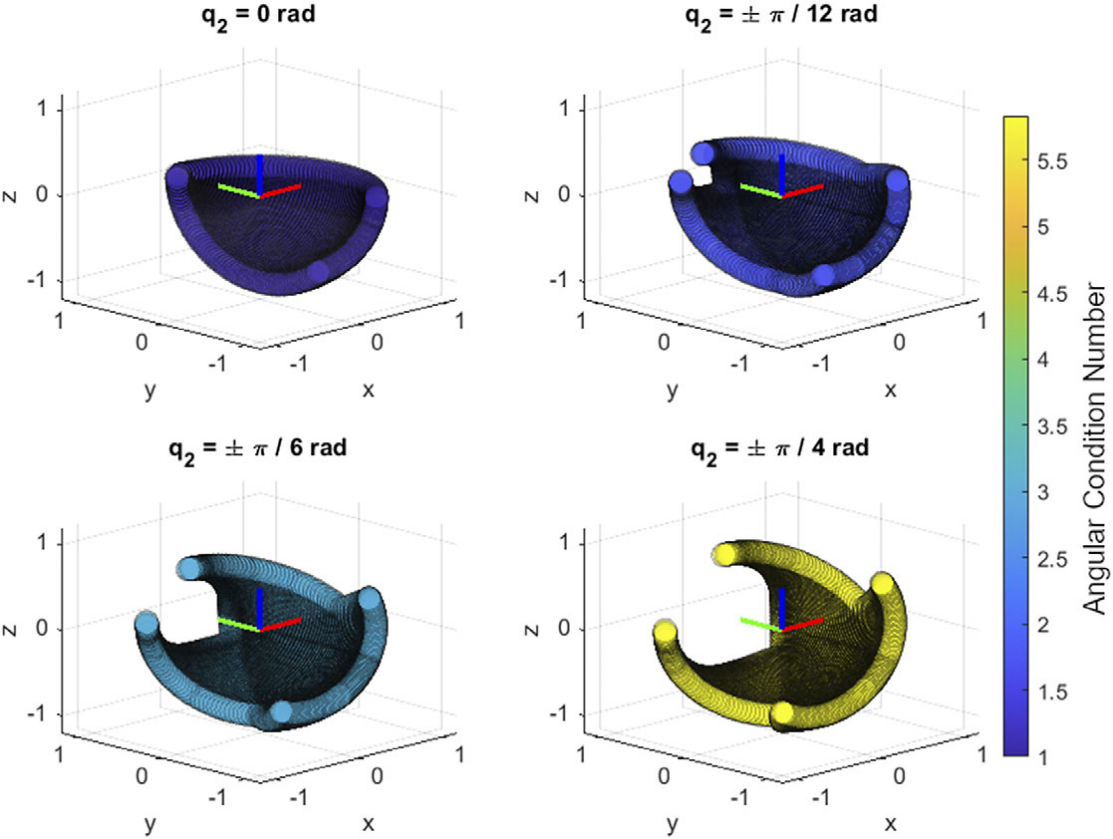
Arm direction as a function of the joint space: The direction of the upper arm is associated with the negative 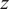 -axis of the end-effector frame. With no passive rotational joint 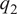 involvement, the arm direction over the full range of 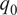 and 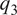 corresponds to a single octant of a sphere and has an angular condition number of 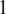 for all values. As 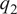 increases, the reachable arm directions cover a larger section of the sphere but the condition number increases.

A scalar metric known as the condition number 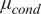 of the angular Jacobian which measures the ratio of the maximum and minimum eigenvalues of the manipulability ellipsoid, written as
(2.13)

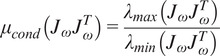

can be computed over the jointspace to show that increasing the angle of 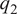 away from 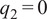 rad is solely responsible for increasing the condition number which lowers the manipulability of the exoskeleton (Figure 3).

### 2.3. Details of the final design

**Figure 4.**
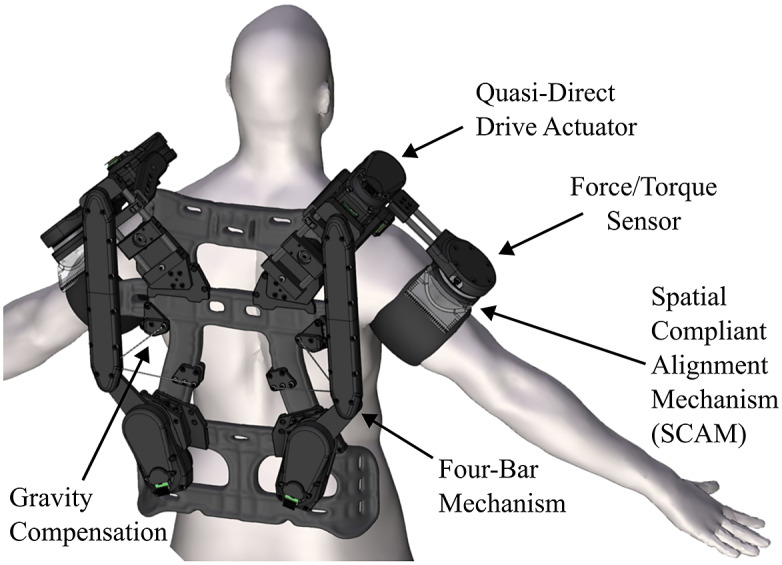
Overview of the mechanical design. The system features a four-bar mechanism to redistribute the weight of the first actuator away from the upper shoulder and keeps the center of mass close to the center torso. The additional space made available by the four-bar allows the inclusion of a gravity compensation mechanism that both fully compensates the weight of robot and partially compensates the weight of the human arm. Finally, a spatial compliant alignment mechanism (SCAM) placed at the interface of the arm and robot allows for passive adjustment of any misalignment between the robot and shoulder center.

The final mechanical design is a straightforward implementation of the proposed kinematic chain along with a few design novelties – namely the addition of a four-bar mechanism for improved weight distribution and gravity compensation, numerically optimized gravity compensation, and the passive multi degree-of-freedom (DoF) SCAM – that allow the relatively simple design to be effective at supporting the complex motion of the human shoulder joint (Figure 4).

The construction of the exoskeleton features many 3-D printed PLA structural components that were carefully designed to balance weight and overall size while not sacrificing structural stiffness. For larger structural components, carbon fiber rods were embedded into the 3-D printed components to provide additional stiffness. Off-the-shelf components such as bearings, drive shafts, and timing belts were included in the design of the quasi-direct drive actuators to provide low friction actuation that would safely transmit the required torques from the motors to the wearer. The final mass of the device was 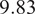 kg with the backpack frame itself being 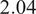 kg of the total.

The motors used were EC 60 Flat 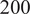 W motors (Maxon Group, Sachesln, Switzerland) with a nominal torque of 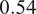 Nm at 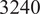 rpm, stall torque of 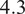 Nm, and rotor inertia of 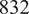 gcm 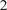 . This was then connected to 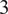 mm pitch GT-3 timing belts (Designatronics, Hicksville, NY, USA) connecting the motor and pulley for a 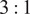 speed reduction. This configuration supports a maximum rated torque of 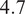 Nm which is below the stall torque after the first stage of gearing. The timing belt then drove a single-stage planetary gear train, resulting in another 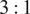 speed reduction for a total reduction of 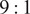 . This gives an output nominal torque of 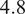 Nm at 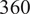 rpm with a stall torque of 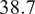 Nm, meeting the specified requirements for the actuator with minimal gearing consistent with quasi-direct drive actuators to allow for backdrivability (Gealy et al., 2019). The motors themselves were equipped with AS5047P absolute encoders (AMS OSRAM Group, Premstatten, Austria) with resolutions of 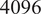 steps per revolution and were controlled with an ODrive 3.6 56 V motor controller which uses a cascaded PID controller to allow simultaneous position and velocity commands to the motors for better responsiveness. Together these drivetrain components create the low friction and highly backdrivable actuators that allow for the motor currents to be used as an estimator for the joint torques, providing redundant sensing of the external force and torque state. The device was connected through a tether of cables to an external power supply, a motor controller, and to an external computer.

### 2.4. Four-bar and gravity compensation

The four-bar mechanism was incorporated into the design to move the actuator for the first active joint from near the shoulder to the wearer’s lower back. This change shifts the exoskeleton’s center of gravity from near the shoulder down to the center of the torso and therefore closer to the human’s natural center of gravity as to not negatively impact the wearer’s balance when wearing the exoskeleton. A secondary benefit of the four-bar mechanism is that it provides ample room to include a dual-purpose gravity compensation mechanism to the system.

**Figure 5.**
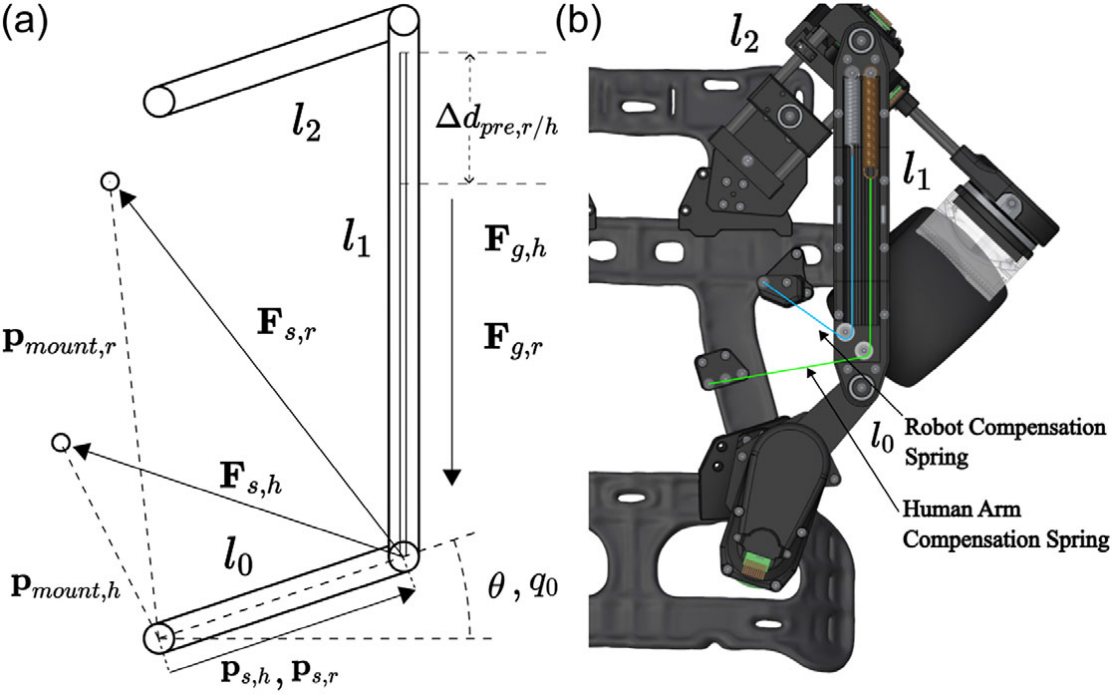
(a) Static model of a four-bar subject to two gravitational loadings (i.e., loadings from the robot and the human arm), 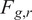 and 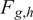 , compensated independently by spring forces 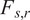 and 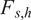 . The physical parameters such as spring rates, initial displacements, and frame mounting locations was found by splitting combined static model into two models and numerically solving an optimization problem to minimize net torques over the four-bar angle 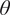 or equivalently 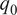 . (b) Physical realization of the results from optimized model with four-bar links 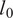 , 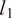 , and 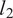 . The springs are placed inside of the main four-bar link and attached cables are redirected by pulleys located near the lower pivot joint and then connected to the frame.

In order to provide gravity compensation for both the robot and the wearer’s arm in a straightforward manner that utilizes the space provided by the four-bar, springs were chosen to be mounted inside of the connecting structure that would be connected to optimized locations on the backpack frame. To determine design parameters such as spring rate and the frame-side mounting locations, a simplified static model of the four-bar was created. This model was derived using the principle of virtual work and considers expected gravity loading from both the robot’s mass and 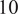 % of the human’s mass as well as the expected torques from the mounted springs as a function of the four-bar angle (Figure 5(a)). The simplified anatomical model used to approximate the human arm’s gravitational torque at the shoulder was based on mass and center of gravity for each segment of a fully extended arm for an average adult human (Plagenhoef et al., 1983):
(2.14)

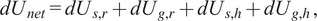

where 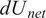 is the net virtual work required to keep the system in static equilibrium, 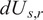 is the virtual work generated by the robot-compensation spring, 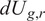 is the virtual work generated by the robot gravitational loading, 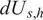 is the virtual work generated by the human-compensation spring, and 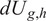 is the virtual work generated by the partial human gravitational loading. The virtual work for the gravitational terms can be computed as
(2.15)

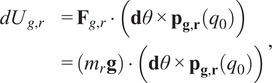



(2.16)

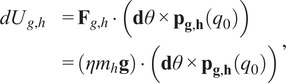

with 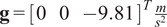 being the gravitational acceleration, 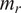 / 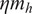 is the supported mass of the robot/human arm represented as a point-mass, 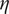 is the percentage of the total human arm mass to compensate, 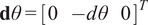 is the differential angle of rotation, 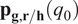 is the position vector from the base of the four-bar to the center of mass of the arm when rotated by 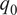 . The virtual work generated by the spring terms can be written as
(2.17)

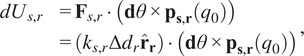



(2.18)

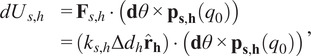

where 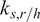 is the spring rate for the robot and human arm compensation springs, respectively, 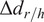 is the net spring displacement from equilibrium, and 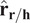 is the line of action of the resultant spring force. The displacement for the robot and human springs can be written as
(2.19)

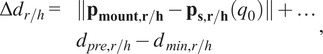

with 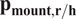 being the mounting locations for the springs onto the frame of the robot, 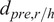 is the initial pre-extension of the springs, and 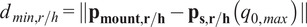 is the minimum distance from the frame-side spring mount and the robot-side spring mounts at the maximum joint angle 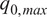 that does not contribute to the net extension of the spring.

As it is desired to have independent compensation for the robot and wearer, this combined model was split into two models with the robot’s gravity torque paired with one compensation spring and the human arm’s gravity torque paired with the other compensation spring (Figure 5(b)).
(2.20)

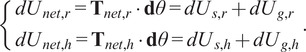



These two models were solved for 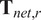 and 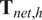 , or the net torques required to statically balance the individual systems, then used to construct a sum of cost functions by evaluating the models with the known robot geometry at evenly spaced 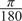 rad increments of the 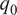 joint angle from 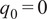 to 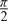 rad, squaring individual terms, and then summing the net torques over the angular domain of 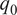 . This process leaves the cost function strictly as a function of the decision variables of 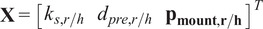 , written as
(2.21)

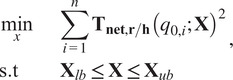

where 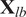 and 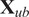 represent the physical constraints such as maintaining a minimum tension on the cable through the minimum pre-extension and spring rates and constraining the area of mounting locations to the backpack frame, and the subscripts 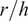 represent the separate robot gravity and human gravity torques to be independently minimized. The optimal spring rates, initial spring pre-extentions, and mounting locations for the given nonlinear cost function were then found by nonlinear optimization available through MathWorks Optimization Toolbox (The MathWorks Inc., 2019) by solving for the parameters that minimize the estimated net torques over the four-bar’s range of motion.

Finally, the four-bar’s effectiveness at lowering the center of gravity closer to a wearer’s center of gravity was evaluated by comparing the real design and an alternative design that removes the four-bar and places the actuator at the top of the exoskeleton where the upper joint of the four-bar is located. The four-bar design lowers the center of gravity by approximately 11 cm and places the robot’s center of gravity near the center of the torso as opposed to near the center of the wearer’s shoulder.

### 2.5. Spatial compliant alignment mechanism

While the passive rotational joint allows for partial misalignment compensation, translation of the shoulder joint along the direction of the upper arm would not be allowed although it can be expected for many natural human movements (Kim and Deshpande, 2017). This second source of misalignment necessitates an additional misalignment compensation mechanism, referred to as the SCAM, for the exoskeleton to passively allow natural human motion. Additionally, this shoulder translation can also generate slight planar misalignment for some arm configurations which would require a multi-DOF alignment mechanism to handle both the translational and planar misalignment.

**Figure 6.**
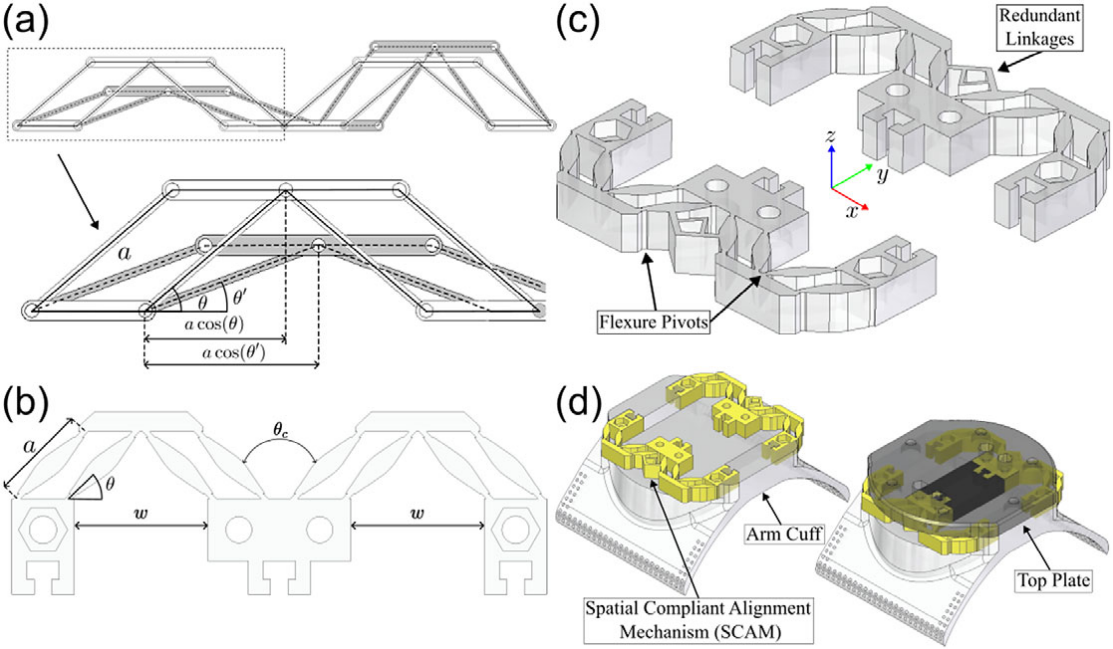
(a) The rigid-body model of the spatial compliant alignment mechanism (SCAM). The design is based on a series of four-bar linkages that allow adjustment of the location of a central platform through a symmetric extension and contraction of surrounding linkages. (b) Compliant mechanism implementation of the rigid-body linkage design. (c) Redundant linkages were added to limit movement via greatly increasing stiffness in undesirable directions by over-constraining the kinematics along those directions while only marginally increasing stiffness along kinematically unconstrained directions. (d) SCAM is mounted between the arm cuff and the top plate and allows relative motion between them along the designed compliant directions.

Since the SCAM must be placed between the flexible fabric arm interface and the mount for the force/torque sensor at the end effector, it is important that the action of the alignment mechanism does not negatively affect the quality of the force transmission from the wearer’s arm to the force sensor (Figure 6(d)). A free-floating slip interface would cause a discontinuous jump in the measured force when the mechanism would reach the end of the range of motion. To address this issue, a compliant mechanism based multi-DOF spring design was chosen and designed by the method of rigid-body replacement (Howell et al., 2013). This method was used to design a conceptual rigid mechanism that would approximate the desired kinematics of the true compliant mechanism. The ideal model resembles a series of connected rigid four-bar mechanisms fixed at the four corners. This allows the center stage to freely translate along 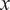 _-_axis through combined extension and retraction of the four-bars links (Figure 6(a)). Using this method, the unconstrained DOFs of the idealized rigid mechanism correspond to low stiffness DOF for the true compliant mechanism and constrained DoF correspond to the high stiffness directions of motion.

To allow translation while maintaining contact with the force sensor, a desired 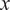 _-_axis translation of the center stage was chosen to be 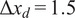 cm. The maximum possible translation without self-collision is 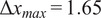 cm and collision is undesirable due to discontinuous jumps in measured forces. To ensure collision is avoided, the rigid link lengths are chosen to be equal to the max displacement, 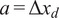 . This causes the extending links to become entirely parallel at the max displacement and the contracting links to retract entirely, stopping any additional movement. The initial angle of the link was chosen to be 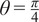 rad to allow equal angular deflection for both extension and contraction of either side of the SCAM. Naturally, this combination of design choices would require a distance of 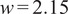 cm between the center stage and either linkage mount which is greater than 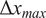 to avoid self-collision (Figure 6(b)). However, the mechanism is intrinsically compliant and was mounted with either linkage mount 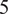 mm closer to the center stage which places the SCAM under compression at the equilibrium.

To convert the rigid-body mechanism model to a compliant mechanism, the rigid links and pivot joints were implemented as flexure pivot joints located along thick compliant links, or blade flexures, that maintain sufficient thickness along ideally rigid sections that suddenly retract in width at areas of high desired flexion at the location of the pivot joints (Figure 6(c)). This design concentrates the bending of the compliant flexure pivots at thin, 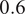 mm sections, although there is slight bending along the 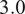 mm blade flexures due to the flexible material. Due to the inaccuracy of a rigid linkage to capture the stretching of flexible material, translation along the 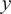 -axis was still possible by allowing both sides of the SCAM to contract simultaneously. To avoid this behavior, an additional constraint of ensuring a constant angle of 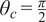 rad between either side of the mechanism was added and implemented by adding redundant linkages to the rigid body model (Figure 6(c)). These redundant linkages do not alter the kinematics of the underlying rigid body model but only serve to improve the approximation to flexible materials by limiting off-axis movement.

The mechanism as well as the arm-cuff that attaches the end effector to the arm were both 3D printed from Cheetah TPU filament (NinjaTek, Manheim, PA, USA), and the cuff itself was sewn to a fabric brace to connect the end effector and the lower arm. This variety of TPU has a Shore hardness of 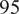 A, a yield strength of 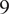 MPa, and an elongation at break of 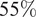 which together make it well suited for repeated loadings with moderate to high deflections.

### 2.6. Controller architecture

**Figure 7.**
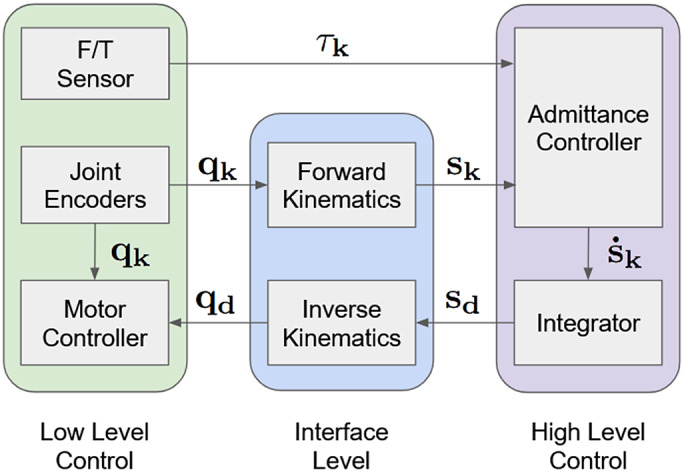
The software architecture for the controls system is divided into three main sections: High level, interface level, and low level. The high-level processes run at 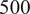 Hz and handle admittance control and the workspace position controller which pass commands through the interface level processes running at 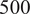 Hz that handle communication to and from external hardware in the low-level processes. The low-level processes handle the commands to the joint level motor controller and communication to external sensors.

For controlling the exoskeleton, a multileveled software architecture was developed that uses the Robot Operating System (ROS) framework to allow for modular control and asynchronous communication between the various required processes (Quigley et al., 2009). The architecture was divided into three key layers, low-level, interface level, and high level; also referred to as Level 0, Level 1, and Level 2, respectively (Figure 7). Level 2, or high level, processes are implemented as ROS nodes and handle tasks such as forward and inverse kinematics, admittance control, and event driven behavior changes in the control scheme which are all running at 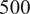 Hz. The interface level processes are implemented as ROS nodes running at 500 or 750 Hz due to differences in sensor sampling rates and communication speeds. The high-level nodes can sample the most up-to-date information from each sensor at 500 Hz. This allows consistent communication between the high-speed low-level processes and the slower high level processes. The low-level processes run on decentralized hardware such as the ODrive motor controller, the Axia-80 force/torque sensor (ATI Industrial Automation, Apex, NC, USA), and microcontrollers that communicate with the passive axis encoder which all run faster than 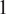 kHz.

### 2.7. Admittance controller

The primary method of controlling the exoskeleton and its interactions with the wearer is through admittance control. Robotic admittance controllers are a special case of force controllers where an ideal dynamical system, typically a second-order mass-spring-damper system, is simulated at the robot’s end effector and is subject to the real measured forces that act to accelerate the system. For this exoskeleton, the basic dynamical model of
(2.22)

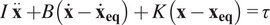

is rearranged into a state space formulation of
(2.23)

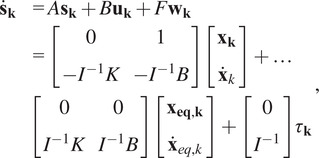

where 
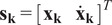
 is the state vector corresponding to fixed frame roll-pitch-yaw Euler angles, 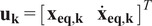 is the controllable input to drive the system toward 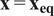 , 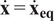 , and 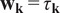 is the uncontrollable input to the system given by the interaction forces and torques between the robot and human wearer, 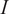 is the simulated inertia or mass, 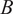 is the simulated damping, and 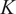 is the simulated stiffness. This system is then discretely integrated in time to generate the desired workspace angular position and velocity commands of 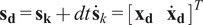 .

## 3. Experimental validation

### 3.1. Gravity compensation through decoupled optimized spring parameters

**Figure 8.**
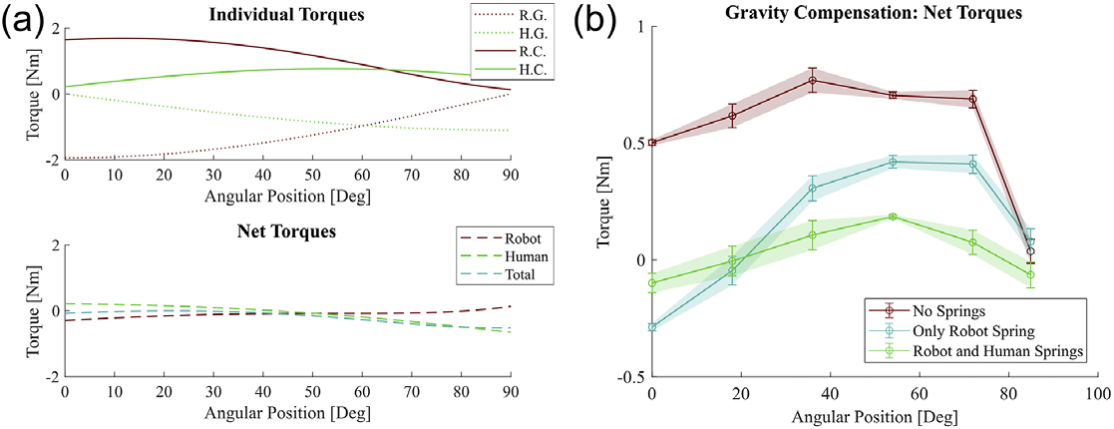
(a) Individual torque profiles for the robot and human arm gravity and compensation torques along with net torques within 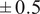 Nm range for both cases. R.G. is the robot gravity torque, H.G. is the human arm gravity torque, R.C. is the robot spring compensation torque, and H.C. is the human spring compensation torque. (b) Motor current based estimated joint torques in real validation study along with a no-compensation spring case for additional reference. In compensated cases, the estimated motor torques are within the expected 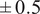 Nm bound and both compensation springs case are within a 0.25 Nm bound over the full angular range of the four-bar.

Results from the static models and optimally chosen compensation springs show the gravitational loading from the robot contributes to a majority of the net torque at low four-bar angles while the gravitational torque from the human is larger at higher angles. With the two independently chosen springs, both can be sufficiently compensated to reduce the net torques close to zero over the entire range of motion (Figure 8(a)). Neither the division of supported torques from either side of the four-bar nor static friction torques can be estimated due to lack of existing research on friction coefficients for the involved materials, but a 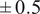 Nm threshold was chosen as a conservative lower bound of the expected static frictional torque and this was shown to be accurate during testing in the following validation study. Therefore, minimizing the net torque to be within this torque range was acceptable as the net torque was less than the torque required to move the exoskeleton.

The springs used in the design that best fit the optimization results were: the robot gravity compensation spring with a spring rate of 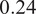 N/mm and maximum extended length of 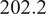 mm (LE 045E 08 M, Lee Spring, AZ, USA) and the partial human arm gravity compensation spring with a spring rate of 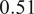 N/mm and maximum extended length of 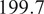 mm (MIL-SPEC MS24586, Lee Spring, AZ, USA).

A validation of the compensation springs’ effect on the net torque was performed by commanding the exoskeleton to hold certain configurations and estimating the torque at the joint through current sensing (Figure 8(b)). The exoskeleton was configured to three cases for comparison: a “No Springs” case with both compensation springs disconnected from the system, a “Only Robot Spring” case where only the spring for supporting the robot’s expected gravitational torque was connected, and finally, a “Robot and Human Spring” case where both supporting springs are connected. For each case, the exoskeleton’s joints were commanded through a joint-level position controller to go to and hold angular positions of 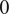 , 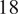 , 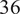 , 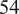 , 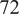 , and 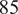 degrees in order to evenly cover the full range of motion. When the exoskeleton reached these configurations, 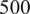 samples corresponding to 1 s of joint current data were recorded and later converted to joint torques and averaged during offline analysis. These trials were repeated 10 times for every case and configuration. The results confirm the compensated net torque to be within the 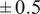 Nm net torque range that was expected through the model, although the uncompensated net torque was measured to be lower than expected. This is likely due to the limitation of the simple model that would result in an overestimate of the expected torques at the active joint that controls the angle of the four-bar, although this limitation does not appear to negatively affect the performance of the finalized system given the recorded joint currents and estimated torques.

### 3.2. Arm to end effector misalignment compensation with SCAM

Experiments were performed to both validate the expected range of motion of the SCAM as well as characterize the stiffness properties. For linear stiffness characterization, the SCAM was mounted to the Axia-80 force/torque sensor and displaced with a power screw in 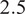 mm increments in a range of 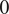 – 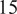 mm while the resultant reaction force was measured for 1 s and then later averaged. For rotational stiffness characterization, the SCAM was similarly mounted to the Axia-80 force/torque sensor and rotated with a power screw in 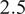 degree increments from 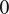 to 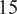 degrees and the resultant reaction torque was measured for 1 s and before averaging. All of these tests were repeated 10 times for every level of displacement and DoF of the SCAM.

**Figure 9.**
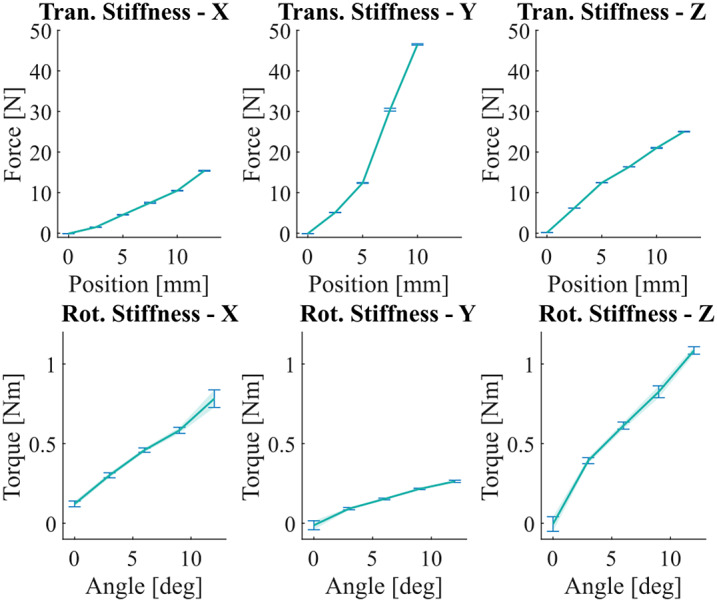
6-DOF SCAM stiffness force profile for all rotational and translation directions. Translational motion along the 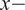 and 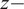 axes and rotational motion along 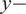 axis are designed to have low stiffness relative to the other directions. These three compliant directions allow for misalignment correction between the arm and end effector by allowing the shoulder center to translate relative to the arm interface and the arm interface to support a combined 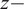 axis translation and 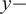 axis rotation to support angular misalignment.

Results from this study show that the expected allowable displacement of 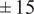 mm was achievable with a smoothly increasing restoring force along the compliant translation directions of 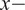 and 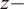 axes (Figure 9). The stiff 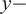 axis translational force response increases rapidly for displacements greater than 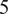 mm and reaches four to five times the stiffness of the 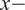 axis. The compliant rotational direction along the 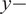 axis shows a similar low stiffness compared to the 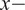 and 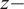 axis directions with stiffness ratios averaging above three times that of the 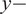 axis for the same angular displacements.

This demonstrates that the planned displacements are not only reachable by the SCAM, but the resultant stiffness force caused by the displacement is sufficiently low to not impede movement. The nonlinear stiffness behavior gives less stiffness force under 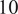 mm of displacement than would be seen with a purely linear spring, but the rapid increase of stiffness force near the end of the range of motion would prevent the user from actually hitting the limit and therefore guide the user back to the center and reduce misalignment.

### 3.3. End effector dynamical system replication with admittance control

To evaluate the exoskeleton’s admittance and position controllers, separate validation experiments were performed that demonstrate the trajectories generated by measured force inputs along with the exoskeleton’s ability to follow the generated motion and show the robot’s ability to reproduce the motion of a simulated torsional spring about an equilibrium angular position.

**Figure 10.**
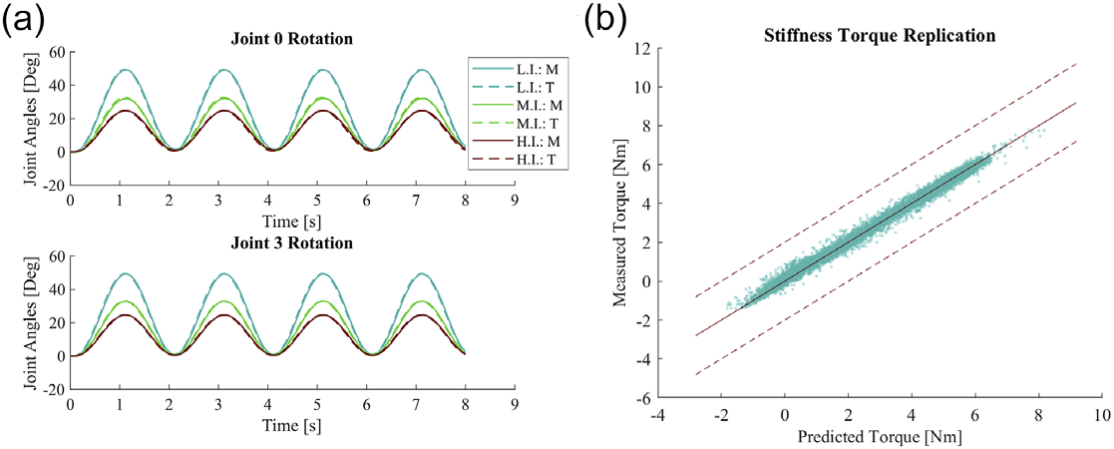
(a) Target and measured angular trajectories generated by the admittance controller subject to a sinusoidal torque input signal for three different simulated impedances: L.I. for low impedance, M.I. for medium impedance, and H.I. for high impedance with T. and M. corresponding for targeted angles and measured angles, respectively. The measured values closely follow the target joint angle values and show that the exoskeleton reproduces the desired dynamical behavior. (b) Measured torques compared to the predicted torques in simulated stiffness tests. The exoskeleton was manually displaced from an equilibrium position and the subsequent measured torques closely follows the predicted torques from a purely simulated dynamical system given the same inputs.

The first validation showed how three different simulated inertial and damping coefficients of the robot follow closely to an ideal system of the same parameters given the same sinusoidal torque inputs (Figure 10(a)). The three cases of 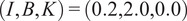 , 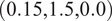 , 
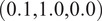
 with units of kgm^2^, Nms/rad, and Nm/rad correspond to “high,” “medium,” and “low” impedances, respectively, while they are all subjected to the same sinusoidal torque input of 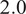 Nm magnitude at 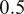 Hz yielding target displacements matching a series of measured displacements (Table 1). The tests were performed with the sinusoidal torque profile firstly along the 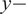 axis, then rotated about the 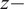 axis by 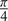 rad, and finally, the initial profile was rotated about the 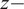 axis by 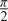 rad. This was done to target different combinations of active joints required to follow the desired profile, with the no rotation or 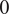 rad case activating only 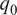 , the 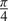 rad case engaging both 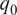 and 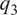 , and the 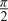 rad case activating only 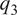 .

**Table 1.** Admittance control replication results

IBK Level Case	Eq. Angle [°]	Joint Axis Label	Target Half angle Range [°]	Measured Half angle Range [°]	Peak-to-peak Difference [°]	Peak time Difference [s]	Joint RMS error [°]
							
Low	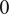	0	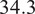	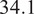	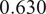	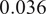	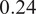
Mid	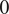	0	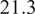	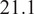	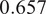	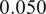	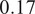
High	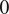	0	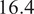	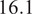	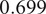	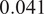	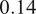
Low	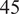	0	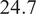	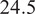	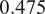	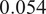	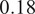
Low	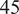	3	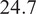	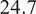	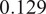	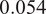	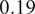
Mid	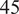	0	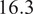	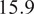	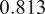	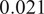	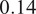
Mid	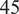	3	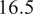	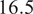	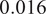	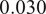	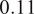
High	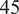	0	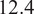	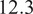	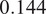	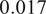	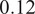
High	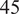	3	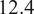	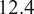	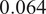	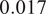	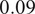
Low	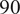	3	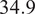	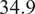	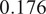	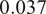	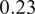
Mid	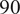	3	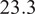	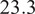	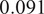	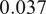	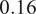
High	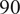	3	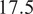	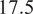	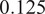	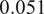	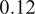

The average peak-to-peak time delay between the target angles and measured angles was 36.4 ms along with a peak-to-peak magnitude difference average of 0.33° with the associated root mean squared error being less than 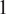 % of the commanded range of motion for all tested cases, suggesting the exoskeleton can follow the ideal dynamical system with sufficiently small error compared to the command signal.

The second validation study attempted to reproduce the robot’s simulated stiffness, damping, and inertia via regression analysis given a series of manual torque inputs in two configurations. The first configuration was with the robot in the origin reference position representing when the wearer is standing with their arm resting to their side, and the second configuration with the arm in a neutral position with the arm in front of the body corresponding to the joint configuration of 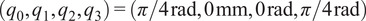 . The exoskeleton’s joint angular position, joint angular velocities, and measured end-effector torques were recorded and fit through a standard least squares regression to the model shown in Equation (2.22)).

The results of the regression are reported and the measured torque due to stiffness is compared to the predicted stiffness torque (Table 2 and Figure 10(b)). The stiffness coefficient was reproduced through linear regression within 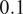 Nm/rad and with percentage variance accounted for ( 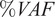 ) above 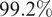 showing consistent stiffness behavior replication for all tested cases.

**Table 2.** Stiffness replication results

Case	Dimension	*K* 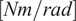	*K*_*est*_ 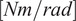	*K*_*std*_ 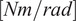	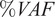
Low 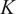 , origin	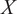	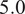	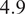	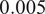	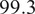
Mid 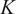 , origin	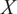	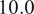	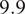	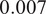	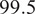
High 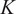 , origin	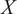	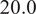	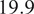	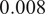	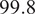
Low 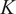 , origin	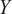	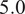	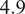	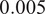	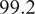
Mid 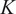 , origin	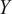	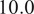	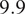	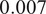	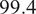
High 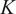 , origin	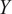	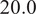	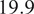	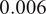	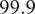
Low 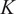 , neutral	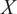	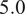	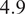	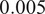	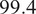
Mid 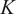 , neutral	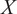	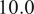	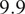	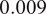	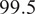
High 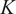 , neutral	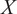	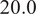	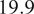	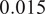	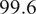
Low 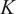 , neutral	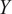	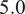	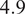	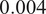	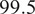
Mid 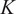 , neutral	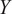	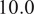	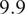	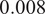	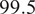
High 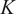 , neutral	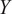	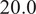	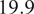	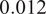	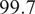

The third validation study was performed to validate the transparency of the admittance controller while simulating low-impedance environments with a human wearer. The subject donned the exoskeleton, and with the admittance controller simulating an impedance of 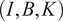 = ( 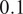 kgm 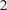 , 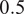 Nms/rad, 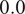 Nm/rad), the subject performed four sets of motions corresponding to common natural movements. These motion cases were a “side raise” where the subject started from standing at rest with their arm at their side to simply raising their arm directly to their side at nearly perpendicular to the floor, a “front raise” where the subject started at rest and then raised their arm directly in front of them, a “neutral raise” where the subject started at rest and raised their arm angled at 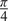 rad from the “front raise” case, and a “neutral reach” case where the subject started with their arm raised 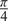 rad to their side and then moved their arm in front of the body similar to the “front raise” case to simulate reaching for an object (Table 3). The motion cases were performed for 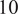 trials timed at 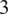 s per motion while measuring the interaction torque between the human and robot. The results show nominal interaction torques less than or equal to 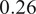 Nm for all motion cases with average peak torques reaching up to 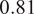 Nm for the “neutral raise” case.

**Table 3.** Transparency study results

Case	Nominal torque (SD) (Nm)	Peak torque (Nm)
Side raise	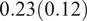	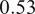
Front raise	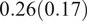	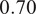
Neutral raise	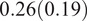	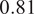
Neutral reach	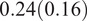	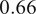

## 4. Discussion

The results from the validation and characterization studies demonstrate how a careful implementation of the chosen straightforward kinematic chain can be augmented by a small number of compensatory design features – specifically, the four-bar mechanism for lowering the center of gravity, the dual gravity compensation for supporting the robot’s weight and partially supporting the wearer’s arm, and the SCAM for mitigating the negative effects of misalignment between the exoskeleton and the wearer – allow for the combined system to function as an effective platform for following and supporting the complex motion of the human shoulder joint.

The experimental validation studies show that each of the major design requirements were satisfied by the prototype. The natural range of motion of the upper arm is reachable by the robot’s end effector. The center of gravity of the exoskeleton is placed near the center of the torso through the addition of the four-bar mechanism which will allow for the user to maintain their natural balance and reduce the risk of lower back injuries. The torque due to gravity from the robot and partial gravity torque from the human arm are compensated by the optimally chosen springs located inside of the four-bar linkage with mounts on the frame. This compensation will reduce fatigue generation in the user’s muscles from the robot’s weight and allows the actuators to dedicate more power to the following and assisting the user rather than supporting its own weight. The misalignment between the kinematic chain’s center of rotation and the moving shoulder center of rotation is reduced by the SCAM located between the arm and the robot’s end effector. The SCAM allows the user to move their arm naturally while also being assisted by the exoskeleton rather than strictly confining the user’s motion to the exact motion of the end effector. Finally, the controlled system was shown to be able to reproduce the behavior of ideal dynamical systems through a responsive admittance controller that is capable of transparent, low simulated impedance operation for a human wearer with nominal interaction torques less than or equal to 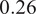 Nm for a sets of common trajectories. Together, these validated requirements show the exoskeleton to be capable of assistance tasks for a wide range of possible cases.

A key limitation in the design is that the full orientation of the end effector is not controllable due to the passive rotational axis 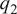 . Ideally, this axis would be replaced with an active axis that passes through the true shoulder center, but this was not feasible due to the required size, self-collision, and weight constraints. Despite this limitation, the exoskeleton does maintain control of the arm’s direction, which is sufficient for supporting and assisting many possible tasks. Additionally, the prototype relies on external hardware connected by a group of cables from the exoskeleton to an external power supply, motor controller, and computer, which limits the range of the exoskeleton. The exoskeleton’s range could be increased by fixing a battery and onboard computational hardware to the lower section of the frame. Finally, the current implementation of the exoskeleton is unilateral with the left side of the device not yet manufactured. This is planned to be addressed simultaneously with design changes to make the device mobile by adding a battery and on-board computing.

The exoskeleton is planned to be used as an evaluation platform to test the effectiveness of assistive controllers based on variations of the standard admittance controller shown in the above sections. The actual assistive performance of the hybrid exoskeleton is dependent on the interaction of the passive features and the active control. Large-scale studies comparing different assistive controllers implemented on this device are planned as future work.

## Data Availability

The datasets generated during and/or analyzed during the current study are available from the corresponding author on reasonable request.
